# *TP53* structural variants in metastatic prostatic carcinoma

**DOI:** 10.1371/journal.pone.0218618

**Published:** 2019-06-19

**Authors:** Deepika Sirohi, Patrick Devine, James P. Grenert, Jessica van Ziffle, Jeffry P. Simko, Bradley A. Stohr

**Affiliations:** Department of Pathology and Laboratory Medicine, University of California, San Francisco, California, United States of America; University of Nebraska Medical Center, UNITED STATES

## Abstract

Sequencing data have been instrumental in identifying oncogenic drivers in prostatic carcinoma and highlighting biomarkers that define aggressive disease. A review of a series of 30 primary and metastatic prostatic carcinomas clinically sequenced at our cancer genomics laboratory utilizing a targeted gene panel identified recurrent structural variants in the *TP53* gene. These structural variants were found in 27% of all sequenced cases and represented 36% of the cases with metastatic disease. *TP53* structural rearrangements have been previously reported in a significant subset of osteosarcomas, where they result in loss of p53 protein expression by immunohistochemistry. Similarly, in our prostate cases with *TP53* structural rearrangements for which tissue was available for testing, we find loss of p53 protein expression by immunohistochemistry. In the eight *TP53*-rearranged cases, concurrent *PTEN* loss was identified in 4 cases, *TMPRSS2-ERG* fusion in 5 cases, and *AR* and *FOXA1* amplification in 1 case each. Our results from this small case series suggest that *TP53* rearrangements with loss of expression represent a frequent alternative mechanism of inactivation of this key tumor suppressor gene with potential utility as a marker of aggressive disease. Recognition of this *TP53* rearrangement pathway is essential to accurately identify prostatic carcinomas with loss of *TP53* function.

## Introduction

Prostate cancer is the second most common cancer in men worldwide [1]. The heterogeneous clinical behavior of prostatic carcinoma complicates treatment decisions and highlights the need for accurate predictors of aggressive disease. Sequencing of primary prostate carcinomas and castration resistant prostate carcinomas have identified recurrent molecular alterations, including *ETS* family transcription factor fusions; mutations in *SPOP*, *FOXA1*, *and TP53; PTEN* loss; and *AR* amplification [2, 3]. Some of these alterations, including *TP53* mutations, are associated with aggressive clinical behavior [4–7].

The function of the p53 protein can be disrupted through a variety of mechanisms, including missense mutations and homozygous loss of the gene locus. Recently, inactivating structural rearrangements involving intron 1 of the *TP53* gene were identified in many pediatric osteosarcomas [8]. Subsequent application of a FISH assay to examine *TP53* intron 1 rearrangements in a wide variety of tumor types suggested that such rearrangements are specific to osteosarcoma [9]. Importantly, these structural variants are not detected by many *TP53* mutation assays, and as a result, it is likely that many osteosarcomas previously considered *TP53* wild-type may in fact be *TP53* mutant [8]. Such misclassification may confound studies examining the impact of *TP53* inactivation on tumor aggressiveness in any tumor type.

Here, based on a small series of cases that underwent targeted clinical sequencing, we report that *TP53* structural rearrangements are frequent in metastatic prostatic carcinoma.

## Materials and methods

Structural variants of *TP53* gene with breakpoints in intron 1 identified in successive cases of prostatic carcinomas prompted a retrospective review of all tumor cases clinically sequenced at University of California, San Francisco (UCSF), with an aim to identify the distribution of pathogenic *TP53* structural variants across different tumor types and specificity for any tumor types. The study was conducted under an IRB (IRB protocol number 15–15823) approved by the University of California San Francisco Human Research Protection Program. Form of consent was not obtained in accordance with the waiver deemed appropriate by IRB as the the data was analyzed anonymously with no more than minimal risk to the subjects. Clinical cases of solid and hematopoietic tumors that included 926 tumors submitted for sequencing over a period of 2 years from 2015 to 2017 were reviewed. Further, we reviewed all *TP53* alterations identified across all cases of prostate carcinomas sequenced. Additional clinical information including sample source, treatment modalities and disease progression to metastatic disease for prostate carcinomas were tabulated. Metastatic designation was defined in accordance with 8^th^ Edition AJCC staging manual and did not include regional lymph node involvement.

**Fig 1:**
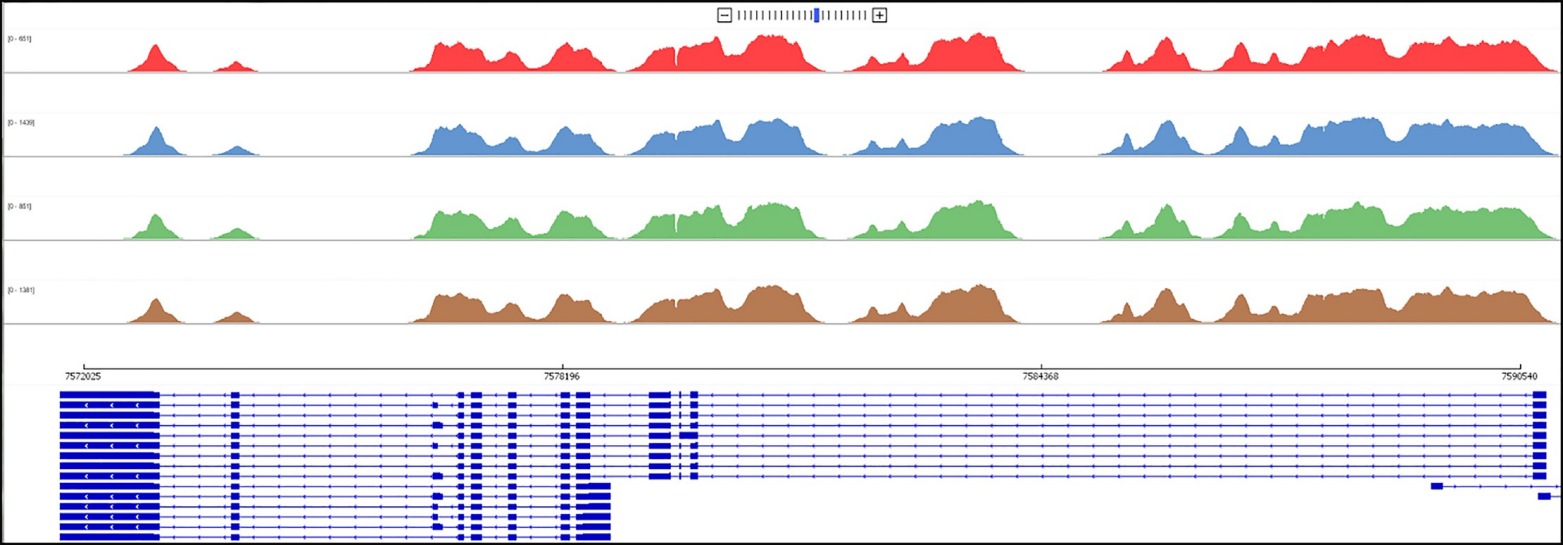
Coverage map across *TP53* gene targeting all coding exons and intron 1, 2, 4, 5, 6, 7, and 8. In intron 1, coverage dips below an average of 10x in two small regions.

Matched normal and tumor tissues were sequenced in all cases. Capture-based next-generation sequencing was performed at the UCSF Clinical Cancer Genomics Laboratory, using an assay (UCSF500 panel) that targets the coding regions of 479 cancer-related genes, select introns from approximately 40 genes, and the *TERT* promoter with a total sequencing footprint of 2.8 Mb as previously described [10]. Structural variants were identified by Delly and Pindel, with verification using the Integrative Genomics Viewer (IGV). All cases were screened specifically for *TP53* gene alterations including missense mutations, small insertions or deletions, copy number changes and structural variants. Specifically, the *TP53* gene being the most frequently mutated gene in cancers is very well covered by the panel, targeting all coding exons and intron 1, with the exception of two small regions of intron 1 where coverage dips below an average of 10x, chr17:7,584,200–7,585,100 (~900 bp) and chr17:7,581,630–7,581,790 (~160 bp), hg19 coordinates. In addition, due to their small size and proximity to targeted exons, introns 2, 4, 5, 6, 7, and 8 are also covered (Fig 1).

Immunohistochemistry for p53 was performed on all cases with available paraffin blocks using standard CLIA-compliant protocols. Immunohistochemical results were broken into 3 categories: overexpression when there was strong nuclear positivity in greater than 90% cells, negative when there was no detectable nuclear staining or wild type when there was variable patchy nuclear staining.

## Results

**Fig 2:**
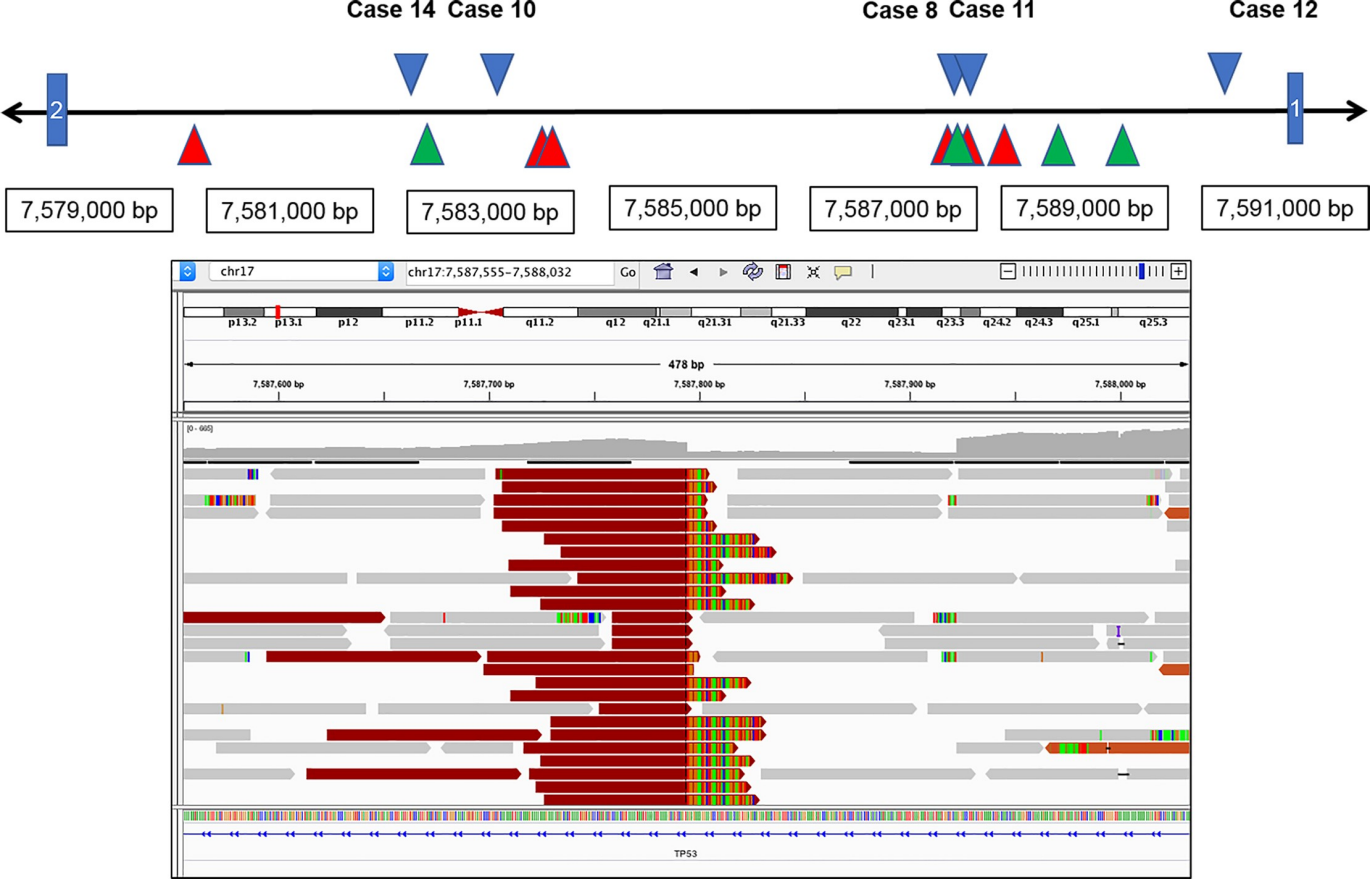
A: Diagrammatic representation of Intron 1 breakpoints in cases of prostate carcinomas with structural variants (blue arrow heads); osteosarcomas (red arrow heads) and other tumors (green arrow heads) B: Reads across breakpoint in intron 1 of *TP53* gene in Case 8 visualized in Integrated Genome Viewer after sorting for insert size. The multicolored reads are unmapped to reference *TP53* genome.

We retrospectively reviewed 926 solid and hematopoietic tumors that were submitted for clinical sequencing over a 2-year period to specifically identify cases with structural variants in *TP53* gene. In all, structural variants involving *TP53* gene were identified in 19 cases (2%). These included 8 out of 30 (26.6%) prostate carcinomas, 5 out of 5 (100%) of osteosarcomas, and 1 or 2 cases of 6 other tumor types (Table 1). While in 12 of these cases, the structural variant identified was a translocation, in the remaining 7 cases, the structural variants were inversions or interstitial deletions. The breakpoints for these variants predominantly involved intron 1 (N = 13) (Fig 2A), and in remaining cases were distributed across exon 1 (N = 2), intron 2 (N = 1), intron 5 (N = 1) and intron 10 (N = 2).

**Table 1:** Distribution of *TP53* fusions across different types.

Tumor type	cBioportal data (11,12)	Current study
Adenocarcinoma, NOS	20% (1/5)	0% (0/35)
Anaplastic astrocytoma	0% (0/141)	5% (1/19)
Anaplastic thyroid carcinoma	3% (1/33)	0% (0/2)
Urothelial carcinoma	0.1% (2/1862)	7% (1/14)
Breast Invasive Ductal Carcinoma	0.03% (2/6205)	0% (0/11)
Cholangiocarcinoma	0.6% (1/150)	0% (0/5)
Colorectal Adenocarcinoma	0.2% (7/3365)	5.7% (2/35)
Cutaneous Melanoma	0.1% (1/977)	0% (0/33)
Leiomyosarcoma	2% (4/205)	0% (0/1)
Lung Adenocarcinoma	0.08% (3/3524)	0% (0/10)
Lung Squamous Cell Carcinoma	0.06% (1/1694)	0% (0/1)
Mixed Germ Cell Tumor	1.5% (1/63)	0% (0/2)
Myxofibrosarcoma	6% (3/50)	0% (0/0)
Oligoastrocytoma	0.36% (1/277)	0% (0/2)
Osteosarcoma	6.9% (3/43)	100% (5/5)
Prostate Adenocarcinoma	0.57% (24/4180)	26.6% (8/30)
Metastatic Prostate Carcinoma	1.27% (14/1095)	33.3% (8/22)
Renal Clear Cell Carcinoma	0.06% (1/1734)	0% (0/20)
Salivary Carcinoma	0.37% (1/270)	0% (0/2)
Serous Ovarian Cancer	0.06% (1/1754)	4% (1/25)
Stomach Adenocarcinoma	0.07% (2/2994)	0% (0/7)
Undifferentiated Pleomorphic Sarcoma/Malignant Fibrous Histiocytoma/High-Grade Spindle Cell Sarcoma	4% (4/99)	100% (1/1)
Uterine Leiomyosarcoma	1.75% (1/57)	0% (0/2)

Of the 926 samples submitted for sequencing, 30 samples were of prostatic carcinoma on which sequencing was performed on primary (N = 15) and metastatic (N = 15) tumor samples and were obtained from patients with metastatic (N = 22) and localized (N = 8) disease. For one case (#15) sequencing was performed separately on both primary and metastatic samples. Of the 15 patients with metastatic samples sequenced, 13 had received radiation therapy, androgen deprivation therapy, and/or chemotherapy, while of the 15 patients with sampling of primary carcinoma, 5 had received radiation therapy, androgen deprivation therapy, and/or chemotherapy. Neuroendocrine features were seen in 2 metastatic carcinoma samples and in 1 primary carcinoma sample. *TP53* gene alterations were identified in 15 of 30 (50%) cases, with 6 of these cases demonstrating missense variants or small insertions/deletions, 8 cases demonstrating structural variants, and 1 case demonstrating homozygous deletion of the gene (Table 2). Of the 15 prostate cancers with *TP53* mutations of any kind, 13 of them had either known metastatic disease at sequencing, or metastatic disease was identified at subsequent follow up. In contrast, of the 15 cases that were *TP53* wild type, 9 had metastatic disease at the time of sequencing or metastatic disease was identified at subsequent follow up; while 6 remained without evidence of metastatic disease on follow up (Table 3). In agreement with prior studies, this suggests that *TP53* disruption is associated with more aggressive disease. However, because our study is a clinical study selected for aggressive tumors, statistical analysis across clinical parameters such as primary and metastatic disease, treatment modalities, tumor grade and histology is not performed. Of note, in one case, we sequenced both the primary and metastatic tumors, and only the metastatic tumor demonstrated the *TP53* rearrangement.

**Table 2:** *TP53* alterations in a subset of prostate carcinomas identified by targeted next generation sequencing and selected clinical features.

Case #	*TP53* alterations	Treatment history	Histologic Features and metastatic site	Sample sequenced	Follow up disease status
1	*TP53* p.F109V	No treatment	Prostate carcinoma, Gleason 4+5, (pelvic lymph nodes)	P	P
2	*TP53* p.F134L	ADT + Radiotherapy	Metastatic prostate carcinoma (spine, liver)	M	M
3	*TP53* p.G334V	ADT + Chemotherapy + Radiotherapy	Prostate carcinoma with neuroendocrine features (bone)	M	M
4	*TP53* p.R196delinsQHLIR	No treatment	Metastatic prostate carcinoma (liver, lung, bone)	M	M
5	*TP53* p.R273C	ADT	Prostate carcinoma, Gleason 5+5, (pelvic lymph nodes)	P	P
6	*TP53* p.Y236C	ADT + Chemotherapy	Metastatic prostate carcinoma (liver, bone)	M	M
7	*TP53* CNV	ADT + Radiotherapy + Proton beam therapy	Prostate carcinoma extending into bladder (rectosigmoid colon)	M	M
8	*TP53* 5' deletion including exon 1, *TP53* rearrangement	ADT + Chemotherapy + Radiotherapy	Metastatic prostate carcinoma (bone)	M	M
9	*TP53* inversion, intron 2	ADT + Radiotherapy	Metastatic neuroendocrine prostate carcinoma (skin, liver, lung)	M	M
10	*TP53* rearrangement	No treatment	Metastatic prostate carcinoma (bone)	M	M
11	*TP53* pericentric inversion	No treatment	Metastatic prostate carcinoma (bone)	P	M
12	*TP53* rearrangement	ADT	Prostate carcinoma, Gleason 4+5 (bone)	P	M
13	*TP53* rearrangement exon 1	No treatment	Prostate carcinoma, Gleason to 4+5 (distant lymph nodes, bone)	P	M
14	*TP53* structural rearrangement with focal deletion	No treatment	Metastatic poorly differentiated neuroendocrine prostate carcinoma (liver)	P	M
15	*TP53* structural rearrangement with focal deletion	ADT + Chemotherapy	Prostate carcinoma with treatment effect (bone)	P	M

Abbreviations: ADT- androgen deprivation therapy, M- metastatic, P- primary.

**Table 3:** Clinical features of *TP53* wild type prostate carcinomas.

	Treatment history	Histologic Features and metastatic site	Sample Sequenced	Follow up disease status
1	ADT + Chemotherapy	Metastatic prostate carcinoma (liver)	M	M
2	ADT + Radiotherapy	Metastatic prostate Carcinoma (lung)	M	M
3	ADT + Chemotherapy	Metastatic prostate carcinoma (testis, brain)	M	M
4	No treatment	Metastatic prostate Carcinoma (bone)	M	M
5	ADT + Chemotherapy + Radiotherapy + Immunotherapy	Metastatic prostate Carcinoma (bone)	M	M
6	ADT + Chemotherapy	Metastatic prostate Carcinoma (bone, lung, liver)	M	M
7	ADT + Chemotherapy + Immunotherapy	Metastatic prostate Carcinoma (bone, liver)	M	M
8	No treatment	Gleason 4+5 (bone)	P	M
9	No treatment	Gleason 4+3 with tertiary 5	P	P
10	No treatment	4+5 with ductal features	P	P
11	No treatment	Gleason 3+4	P	P
12	No treatment	Metastatic prostate Carcinoma (spine)	P	M
13	No treatment	Gleason 3+4	P	P
14	ADT + Chemotherapy	Gleason 4+4	P	P
15	Immunotherapy	Gleason 4+5	P	P

Abbreviations: ADT- androgen deprivation therapy, M- metastatic, P- primary.

The structural variants in 8 cases of prostatic carcinoma included breakpoints in intron 1 (N = 5), intron 2 (N = 1), exon 1 (N = 1) and intron 10 of *TP53* (Fig 2) and are predicted to result in loss of gene expression. Amongst the structural variants, 5 were translocations with different fusion partners (namely *DNAH2* (2 cases), *HDAC9*, *PACS1* and *TMEM107*) while 3 were inversions (Table 4). Additionally, 2 of the cases had concurrent exon 1 deletions.

**Table 4:** Structural variants in prostate carcinoma.

Case #	Type of Structural Variants	*TP53* Breakpoint	Partner Gene
8	*TP53* translocation and 5' deletion including exon 1	Intron 1	*DNAH2* (Chr 17) intron 40
		Exon 1 deletion	Not applicable
9	*TP53* inversion	Intron 2	Intergenic
10	*TP53* pericentric inversion	Intron 1	Upstream of *CBX8*
11	*TP53* rearrangement	Intron 1	*TMEM107* (Chr 17) exon 3
12	*TP53* rearrangement	Intron 1	*DNAH2*
13	*TP53* rearrangement	Exon 1	*PACS1*
14	*TP53* structural rearrangement with focal exon 1 deletion	Intron 1	*HDAC9* (Chr 7) intron 11
		Focal exon 1 deletion	Not applicable
15	*TP53* inversion	Intron 10s	Intergenic

**Fig 3:**
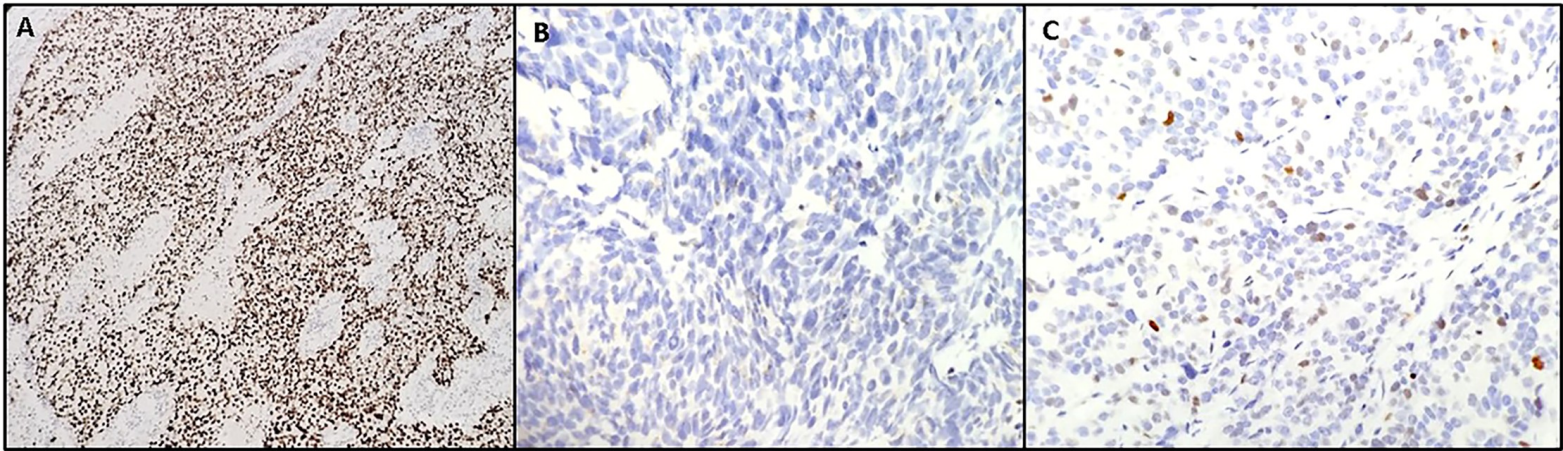
p53 immunohistochemical expression pattern in prostate carcinomas with *TP53* alterations. A- Diffuse nuclear positivity (> 90%) is seen in a case with *TP53* p.R273C mutation (10x). B- *TP53* structural rearrangement resulting in complete loss of *TP53* expression. Staining of background stromal cells and inflammatory cells seen as an internal control (20x). C- Prostate carcinoma with wild type *TP53* showing weak and patchy (less than 5%) nuclear staining (20x).

Immunohistochemical analysis for p53 protein expression was done for 15 cases with available blocks; these included 3 cases with structural variants (all metastatic carcinomas), 3 with pathogenic missense variants (1 primary, 2 metastatic carcinomas), and 9 with no detectable *TP53* alterations (4 primary, 5 metastatic carcinomas). As expected, all 3 cases with structural variants showed a complete absence of detectable nuclear staining, consistent with loss of protein expression, while the 3 cases with pathogenic missense variants showed strong nuclear positivity in more than 90% of the cells. In the 9 cases lacking detectable *TP53* alterations, the staining ranged from 5 to 50% with variable nuclear staining intensity (Fig 3).

Other genetic alterations identified in cases with structural variants (all metastatic carcinomas) included *TMPRSS2-ERG* fusions in 5 of 8 cases, *PTEN* copy number loss in 4 of 8 cases (homozygous deletion of the entire gene in 3 cases and 18-bp deletion of intron 1 case), and *AR* and *FOXA1* amplification in 1 of 8 cases each. Of the 7 prostatic carcinomas with missense mutations, small insertions/deletions and copy number changes in *TP53*, *TMPRSS2-ERG* fusion was seen in 4 cases, *PTEN* mutations/ copy number loss in 3 and *AR* amplification in 1.

## Discussion

The *TP53* tumor suppressor gene is amongst the most frequently mutated genes in human cancers. Most mutations in *TP53* are single nucleotide variants or small insertions/deletions resulting in missense, nonsense, truncating, splice site and frameshift alterations [11, 12]. Structural variants have been reported much less frequently in osteosarcomas, prostate carcinomas, small cell lung cancer [8, 9, 13–16, 17] and more recently on deep whole genome analysis of castrate resistant metastatic prostate carcinomas [18]. Here, based on a small series of cases undergoing targeted sequencing for clinical purposes, we have shown that *TP53* structural rearrangements are an unexpectedly common cause of *TP53* inactivation in advanced prostatic carcinomas.

*TP53* structural rearrangements involving intron 1 were initially reported in the context of osteosarcomas [15, 16]. More recently, using whole genome analysis of 52 osteosarcoma samples, Chen et al [8] found clonal *TP53* structural variants in 55% of cases, 90% of which had breakpoints in intron 1. *TP53* structural variants have also been reported in osteosarcoma cell lines [15], rare instances in myeloid leukemia [18, 19] and blast crisis in chronic myelogenous leukemia [20], and in the germline of some families with Li- Fraumeni syndrome [9]. FISH analysis of 215 osteosarcomas using probes directed at the *TP53* gene found biallelic structural rearrangements in 11% of cases [9]. In contrast, the FISH test did not identify *TP53* structural rearrangements in other 124 bone forming tumors and tumor like lesions or in 966 other tumor samples, including 33 prostatic adenocarcinomas. Based on these FISH results, the authors suggested that such *TP53* intron 1 rearrangements may be specific to osteosarcomas. However, in their study, the authors did not provide additional details of whether these were primary or metastatic prostate carcinomas and the tumor grade that could account for the differences in detection from the current study.

In contrast, our data suggests that in addition to osteosarcoma, inactivating *TP53* rearrangements involving intron 1 are quite common in prostatic carcinoma. We identified *TP53* structural variants in 8 of 30 (27%) prostatic carcinomas. While these structural variants were seen in primary and metastatic tumor samples, all cases progressed to metastatic disease. Thus, 36% (8/22) of the patients with metastatic disease in this small cohort demonstrated *TP53* inactivation through structural rearrangement. Most frequently the breakpoint occurred in intron 1, similar to prior reports in osteosarcomas [9] and prostatic carcinomas [14], with a few breakpoints elsewhere in the gene. The immunohistochemical expression of p53 protein also correlated with underlying molecular alterations as also demonstrated in other studies. Both high levels of expression or complete absence of staining have been shown to correlate with mutant *TP53* status [21–23], suggesting the use of the immunohistochemical stain as a good marker for mutant status of the gene, although the staining pattern is not specific for the mutation type. Pathogenic mutations in *TP53* result in either loss of p53 expression or its ability to bind to DNA response elements. A subset of *TP53* mutations result in gain of oncogenic function or mutations with dominant negative effect with accumulation of mutant protein at high levels [24] and some of these have also been associated with development of chemoresistance [25].

In general agreement with our findings, examination of the cBioportal database [11, 12] demonstrates *TP53* fusions in 68 out of 65,690 samples queried (0.1%), including 24 of 4365 samples (0.6%) of prostatic carcinomas from 4180 patients (accessed 8/14/2018), with prostate carcinomas being the most common tumor type with *TP53* fusions (24/68) (Table 1). Specifically in the metastatic prostate carcinomas, *TP53* fusions were reported in 14 of 1095 cases (1.27%). The fusions were distributed across 10 primary prostatic carcinomas and 14 metastatic prostatic carcinomas. *PTEN* alterations, *TMPRSS2-ERG* rearrangement, *AR* alterations and *FOXA1* mutations were identified in 7, 17, 8 and 3 of the 24 cases respectively. The *TP53* rearrangements reported in cBioportal are intragenic fusions or translocations, all involving different fusion partners. There may be several reasons for the lower rate of detection of *TP53* fusions in cBioportal database including potentially different grades of tumors analyzed, intronic coverage that could be significantly less on whole genome or whole exome sequencing as compared to more targeted sequencing for clinical assays, and lack of bioinformatic support for detection of these alterations.

Recently whole genome analysis of 57 primary prostatic carcinomas and transcriptome sequencing of 20 primary prostate carcinomas [13] identified numerous interdependent translocations and deletions occurring through a process of concurrent disruption of several genes in a coordinated manner that the authors termed chromoplexy. Resultant gene disruptions involved spatially separated genes as well as genes in the same pathway, affecting multiple cancer genes. Oncogenic genes with recurring deletions or rearrangements in their study [13] included *PTEN* (N = 9), *NKX3-1* (N = 8), *CDKN1B* (N = 3), *TP53* (N = 4) and *RB1* (N = 2). Clonal evaluation of altered genes led to a proposed oncogenic model of cancer progression initiated by deletion of *NKX3-1* or *FOXP1* and *TMPRSS2-ERG* fusion, followed by *CDKN1B* or *TP53* alterations and finally ending in *PTEN* loss. More recently, deep whole genome analysis of castrate resistant metastatic prostate carcinoma also identified structural variants in tumor suppressor genes including *TP53*, *PTEN*, *RB1*, *CDKN1B* and *CHD1* resulting in biallelic gene inactivation, novel gene fusions and tandem gene duplications. In this analysis, biallelic inactivation of *CDK12*, *BRCA2* and *TP53* strongly correlated with the structural variants and chromothripsis [18].

The higher percentage of *TP53* structural variants found in our study may be a reflection of the small study size and relatively high percentage of metastatic tumors in our cohort. Tumors chosen for sequencing tend to be aggressive in nature, as a frequent goal of sequencing is to find additional targetable alterations in advanced cases. It is unclear if *TP53* rearrangements will be found in any significant number in lower grade organ confined disease. We recommend prospective studies to evaluate for distribution of *TP53* structural variants in primary and metastatic prostatic carcinomas, which might serve as a marker of aggressive disease and disease progression when detected.

The primary limitation of our study is its small size, which limits our ability to determine the clinical significance of the *TP53* rearrangements identified. Moreover, the limited sampling could lead to over or underrepresentation of the various *TP53* alterations identified in our cohort.

## Conclusion

In this small series, we report the occurrence of *TP53* structural variants in a significant subset of metastatic prostatic carcinomas that underwent targeted sequencing for clinical purposes. Recognition of this alternative mechanism of *TP53* loss of function is important to properly characterize the genetics of prostatic carcinomas for both clinical and research purposes, as some assays will not detect these structural rearrangements. Our findings need to be validated in a larger cohort of metastatic prostate carcinomas.
